# Signs, symptoms and diagnosis of necrotizing fasciitis experienced by survivors and family: a qualitative Nordic multi-center study

**DOI:** 10.1186/s12879-018-3355-7

**Published:** 2018-08-28

**Authors:** Annette Erichsen Andersson, Ingrid Egerod, Vibeke E. Knudsen, Ann-Mari Fagerdahl

**Affiliations:** 1 Institute of Health Care Sciences, Sahlgrenska Academy, University of Gothenburg, Box 457, 405 30 Göteborg, Sweden; 2 Department of Orthopaedic Surgery, Sahlgrenska University Hospital/Mölndal, Gothenburg, Sweden; 3 Faculty of Health and Medical Sciences, University of Copenhagen, Copenhagen, Denmark; 4 Rigshospitalet, Copenhagen University Hospital, Intensive care unit 4131, Blegdamsvej 9, 2100 Copenhagen, Denmark; 5 Wound Centre, Södersjukhuset, Department of Clinical Science and Education, 118 83 Södersjukhuset, Karolinska Institutet, Stockholm, Sweden

**Keywords:** Necrotizing soft tissue infection, Patient experiences, Family perspective, Qualitative study, Diagnosis, Signs and symptoms

## Abstract

**Background:**

Necrotizing soft tissue infection is the most serious of all soft tissue infections. The patient’s life is dependent on prompt diagnosis and aggressive treatment. Diagnostic delays are related to increased morbidity and mortality, and the risk of under- or missed diagnosis is high due to the rarity of the condition. There is a paucity of knowledge regarding early indications of disease. The aim of the study has thus been to explore patients’ and families’ experiences of early signs and symptoms and to describe their initial contact with the healthcare system.

**Methods:**

A qualitative explorative design was used to gain more knowledge about the experience of early signs and symptoms. Fifty-three participants from three study sites were interviewed. The framework method was used for data analysis.

**Results:**

Most of the participants experienced treatment delay and contacted healthcare several times before receiving correct treatment. The experience of illness varied among the participants depending on the duration of antecedent signs and symptoms. Other important findings included the description of three stages of early disease progression with increase in symptom intensity. Pain experienced in necrotizing soft tissue infections is particularly excruciating and unresponsive to pain medication. Other common symptoms were dyspnea, shivering, muscle weakness, gastrointestinal problems, anxiety, and fear.

**Conclusion:**

Our study adds to the understanding of the lived experience of NSTI by providing in-depth description of antecedent signs and symptoms precipitating NSTI-diagnosis. We have described diagnostic delay as patient-related, primary care related, or hospital related and recommend that patient and family narratives should be considered when diagnosing NSTI to decrease diagnostic delay.

## Background

Necrotizing soft tissue infection (NSTI) is the most serious and potentially life-threatening of all skin and soft tissue infections and prompt diagnosis and aggressive treatment are of utmost importance to save patient lives [1]. NSTIs include necrotizing cellulitis, necrotizing fasciitis, Fournier’s gangrene and necrotizing myositis [2]. The condition is rare with incidence ranging from 0.4 to 7.7 per 100,000 [3–6]. Several studies have shown a rise in incidence over the past decades [3, 4, 7, 8].

NSTIs typically involve all the soft tissue compartments from skin to the deeper fascia and muscles. NSTIs affect most parts of the body, particularly the extremities, perineum and abdominal wall. The disease is generally caused by microorganisms such as streptococci and staphylococci that are found on skin and mucosa on healthy individuals. The causative pathogens attack the subcutaneous tissues and produce toxins causing ischemia, necrosis and septic shock that eventually lead to systemic organ failure [1, 3]. The course of the disease is described as rapid, leaving the patient to deteriorate within hours if not treated correctly. Rapid, aggressive treatment with surgical debridement and antibiotic therapy is lifesaving, whereas treatment with antibiotics alone might be ineffectual due to tissue necrosis. Additional therapies are given depending on causative agents, location and type of infection [9]. More than 50% of the patients require intensive care and mechanical ventilation, central line catheters and hemodialysis [3, 10] as systemic complications and organ failure are common (40–60%) [3, 11].

Mortality is high despite improved treatment and care (10–29, 3%) [3, 10–13]. NSTI can be seen in patients of all ages, with or without comorbidities [14]. Conditions that have been associated with necrotizing fasciitis include: diabetes, obesity, renal disease, smoking, alcohol abuse and immunosuppression [1, 3, 13, 15]. Patients can acquire NSTI in several ways: the pathogens can be inoculated into the subcutaneous tissues via injections, trauma, animal bites, surgical interventions, skin infections and childbirth, and probably also via hematological spread in cases with suspected streptococcal pharyngitis [2, 16]. However, it is not uncommon that no definitive cause can be found [1, 17].

NSTIs can be described according to anatomical location or type and number of pathogens involved [18]. Typically, 3 classes of pathogens are described in the literature [2, 16]. Typical signs and symptoms of NSTI are first of all pain, with other responses including localized edema, erythema, fever, hypotension, perspiration, skin necrosis and crepitus [1, 13]. In the early stages, the risk of under-diagnosis is high since the condition is rare. For example, one study demonstrated that only 14% of patients with NSTI were properly diagnosed on admission [17]. Delays in diagnosis increase the rates of morbidity and mortality [9, 19]. Studies are still lacking that describe early signs and symptoms of NSTI from the perspective of patient and family. This type of study can potentially contribute new knowledge concerning the onset and dissemination of NSTIs. The aim of our study was to explore the patient and family experience of early signs and symptoms of NSTI in NSTI survivors and to describe their initial contact with the healthcare system, in order to delineate strengths and weaknesses in care provided.

## Methods

### Study design

The study is embedded in a larger EU study, the INFECT-study, aiming to understanding the pathophysiology of NF and other NSTIs. As a contrast to the INFECT-study, we chose in the present study (the P-INFECT-study) to apply a qualitative explorative design to gain more knowledge about the experience of early signs and symptoms of NSTI in patients and their families [20]. From the onset, the study was planned and designed in close cooperation with NSTI survivor Kim Andersson and his wife (patient and public involvement). The present study is the third in a planned series of five qualitative studies investigating NSTI from the perspective of patients and their families (ClinicalTrials.gov Identifier: NCT02169128). Our first paper provided a template to inform the patient and family of a typical NSTI-trajectory and what to expect [20]. Our second paper described family caregivers’ coping strategies in ICU, in the ward, and after returning home [21].

### Participants and selection

Patients and families were recruited from three centers where treatment of NSTI was centralized a national level in Sweden and Denmark. In the INFECT study, NSTI was confirmed by perioperative tissue characteristics, observed by the surgeon. Diagnosis was based on signs of necrotic soft tissue with widespread undermining of the surrounding tissue [22]. In the present study, the P-INFECT study, we screened discharged patients with confirmed NSTI for eligibility using the following criteria: NSTI diagnosis, age ≥ 18, and provision of written informed consent to participate in the study. Exclusion criteria were dementia or severe psychiatric illness.

All participants were provided with written and verbal information regarding this qualitative study in relation to the INFECT study. Close family was defined by the patients and included husband/wife, partner, sibling, children and close friend. At 6 months after diagnosis the participants received a letter with study information and we asked both patient and family to confirm that they agreed to be interviewed. After confirmation, the participants were contacted by phone to decide on the location for the interview. The participants were included consecutively, however the intention was to achieve a heterogeneous group in terms of age and sex in order to obtain as rich data as possible. It was estimated that approximately 10 patients and 10 close family members at each study site would be an appropriate sample size to ensure variation of the phenomenon in focus and to achieve the intended purpose [23]. Data saturation was assessed during data collection to determine the final sample size.

### Context of the study

The healthcare systems in Sweden and Denmark are funded by taxes. In Sweden the patient pays approx. EUR 30 for an emergency room visit and EUR 20 for a visit to the General Practitioner, GP. There is no charge in Denmark. Primary service centres and hospitals provide multidisciplinary care, and out-of-hours (OOH) service is available to handle urgent and emergent illness when the GP is unavailable. OOH call handlers provide immediate support to the caller and triage the patient to the type of service needed.

### Data collection

Data were collected from August 2014 to March 2016 using in-depth semi-structured individual interviews to allow the participants’ experiences of and perspectives on NSTI, care and treatment to emerge [24]. After consulting our patient representative Mr. Andersson and family, we decided to conduct the interviews 6 months after diagnosis. This would allow the participants to come to terms with the initial shock of diagnosis and early treatment. We preferred to conduct the interviews face-to-face, but if this was not feasible some interviews could be conducted by telephone. The interviews were carried out by investigators with experience of caring for patients with NSTI in the acute setting.

Our main question was: *“Please tell me: how did you experience your illness?”* To ensure that we understood the participants’ responses, we posed clarifying questions during the interview. If necessary, additional questions were asked regarding early signs and symptoms and the first contacts with healthcare. All interviews were digitally recorded and transcribed verbatim. Demographic data were obtained from medical charts.

### Data analysis

The interviews were analyzed using the framework method [25] that allowed for the development of themes from the narratives of the participants as well as research questions. The analytic process involved five interrelated key stages, see Table 1. Several measures were taken to ensure the trustworthiness of our study [26] (chapter 9). Tentative themes were cross-validated by 25% of the informants during a post-interview meeting. To enhance creditability, the final themes and sub-themes were challenged by searching data for alternative themes, divergent patterns and rival explanations. In addition, findings were discussed between all the researchers until consensus on interpretation was reached. To enhance credibility, the results are supported by representative quotes.

**Table 1:** Analytical steps in the Framework method

Analytical steps	Our process of analysis
Familiarization	All researchers read transcripts and listened to tape recordings to become familiar with the data.
Identification of a thematic framework	Two researchers independently coded the first transcript before developing a tentative coding framework using a priori research questions and emergent issues. All researchers contributed feedback and adjustments.
Indexing	The thematic framework was applied to each transcript and the texts were coded accordingly, e.g. signs and symptoms, etc.
Charting	Matrixes were created for every key subject area. Data from each case and code were then charted within the themes.
Mapping and interpretation	Each matrix was analyzed inductively and data were compared to detect similarities, differences and patterns.

## Results

### Demographics

We conducted 53 interviews at the three study sites: 10 patients and 9 family members in Gothenburg (SU), 8 patients and 6 family members in Stockholm (KI), and 10 patients and 9 family members in Copenhagen (RH). Thus, we interviewed 28 patients, 18 men and 10 women 29–90 years of age. The infection was located in their extremities (*n* = 16), groin/genitals and lower abdomen (*n* = 7), face and throat (*n* = 3), and back (lateral dorsi) (*n* = 2). Tissue samples showed growth of different types of streptococci, Group A being the most prevalent (*n* = 22) followed by groups G and B. Poly-microbial infection was found in 7 cases including growth of *Staphylococcus aureus* and *Staphylococcus epidermis.*

### Qualitative findings

Our analysis covered the time from initial signs and symptoms to diagnosis of NSTI. We identified three themes describing the experience of patient and family in the early stage of illness as shown in Table 2: Duration of disease onset, Stages of symptom severity, and Types of treatment delay.

**Table 2:** Themes and sub-themes

Themes	Sub-themes
Duration of antecedent signs and symptoms	Long inception: More than 7 days Moderate inception: 3–7 days Short inception: 1–2 days
Stages of symptom severity	Stage I. Lingering symptoms Stage II. Progressive symptoms Stage III. Unbearable symptoms
Types of diagnostic delay	Patient-related delay Primary care related delay Hospital related delay

### Duration of antecedent symptoms

The experience of illness varied among the participants in our study depending on the perceived duration of disease onset from long to short inception. We defined **long inception** as a period of more than 7 days with gradual escalation of antecedent signs and symptoms before life-threatening diagnosis. This was often associated with a month of malaise with recurrent streptococcal throat infections at home or at work, perhaps increasing the patient’s susceptibility for serious disease. **Medium inception** lasted 3–7 days, with signs and symptoms gradually emerging and increasing in intensity. **Short inception** lasted 1–2 days, with antecedent signs and symptoms rapidly progressing to severe impairment of the patient’s mental and physical state. In our sample,11 patients experienced a short inception, 9 described a moderate inception, 6 described a long inception, and one was undetermined.

#### Case description of long inception

A otherwise healthy person experienced a sore throat recurrently during early spring and felt unwell for some weeks with a fluctuating temperature:

*“In the beginning of May I felt increasingly crummy. I couldn’t exactly describe what was wrong but there were no signs on my body that indicated what was wrong”.*

The last 2 weeks before diagnosis the person felt increasingly worse and finally the symptoms became so intense that they could no longer be ignored:

*“Then one day at the end of May I felt that my body had no more resistance … it was like a really bad case of the flu, and my leg started to ache”.*

The pain increased and finally became unbearable: *“much worse than being in labor”*. Her husband drove her to the emergency department (ED), where she was finally assessed and treated. At this time, person was confused and dizzy with pain:

*“After blood tests, they diagnosed me with severe sepsis and I have read my chart saying that just 2-3 hours later, I suffered total organ failure”.*

#### Case description of moderate inception

On Monday, a man started to feel poorly. On Wednesday he woke up in the morning with a sore throat and contacted the medical on-call service. The call handler advised him that he was okay if he had no white spots in his throat. On Thursday, the throat was more painful and he had a hard time swallowing painkillers, but still no visible white spots. On Saturday he ran a fever and felt pain in his thigh. He was unable to swallow and needed help. His wife contacted the medical on-call service and the call handler recommended paracetamol (acetaminophen). After a few hours, his wife called again because the medicine gave no relief and they both feared that something was terribly wrong. His wife made a doctor’s appointment and tests revealed strep throat with elevated levels of C-reactive protein (CRP). He complained to a nurse of pain in his thigh, but the nurse said not to worry, because there was no swelling. He returned home with a prescription for antibiotics. On Sunday his throat felt better, but the pain in his leg had gotten worse:

*“I had a really bad feeling, it was scary and I felt that I no longer had control over the situation”.*

He became nauseated and was unable to move due to severe pain in his leg. He contacted the medical on-call service and got a doctor’s appointment. At this point his thigh had swollen rapidly over the course of few hours. A new set of tests showed a CRP over 200 and he was sent to the hospital. An ambulance was not dispatched at this time:

“*I had to almost carry him into the car. At the hospital, they immediately understood that something was wrong and we were both taken care of”.*

#### Case description of short inception

A woman was relaxing in her garden after a day at the beach. Suddenly she felt pain in her lower leg, and shortly after the leg turned red. Within a few hours of the first signs and symptoms, the pain and discomfort escalated and she asked her son to help her to bed. Her son contacted the medical on-call service and was advised to give ibuprofen and wait: *“It will pass*”. The pain became unbearable and the medical on-call service was contacted another two times:

*“I panicked, I have never experienced such pain before, I asked for an ambulance, but the doctor said: ‘do you really want to burden the healthcare system with the extra expenses?’”*

In the evening she had deteriorated to the point that the family decided to take her to the nearest hospital:

*“At first they said there was nothing wrong with me – all the blood tests were okay. I was placed in the hallway of the hospital when an orderly from the ED discovered something was very wrong. He quickly got me a physician…I don’t remember anything after that”*

### Stages of symptom severity

The narratives from patients and their family members provided a good picture of the disease manifestations. We present these in three stages of symptom severity that appear differently depending on the duration of disease onset. In **the first stage** the patients described flu-like symptoms that could last for a month during long inception. *“My muscles were sore like after exercise”*. In **the second stage,** most patients and family started to feel that “*something was seriously wrong”.* The spectrum of symptoms included pain, malaise, shivering, fever and nausea. The intensity of pain escalated and most of the patients became bedridden due to muscular weakness. Patient and family became anxious as the disease felt life-threatening: *“It was a scary feeling of losing control”… “I felt really bad and started to get the feeling that I might die”.* The patients became so weak that they were unable to move about. Both patients and their families realized that something was wrong. **The third stage** included intensified pain, fear and anxiety with confusion, dizziness or fainting. Memory of this stage was often fragmented although some patients were able to recount what had happened: *“The nurse couldn’t measure my blood pressure and said that there must be something wrong with the machine”… “At the ED the doctor cut my arm … I couldn’t feel this even though I was not anesthetized … my arm was so swollen”.* One patient said that when her condition was finally taken seriously, the last thing she recalled before passing out was the nurse calling for adrenaline and the doctor compressing the fluid bags.

The signs and symptoms of infection described by the patients are displayed in Table 3. Symptoms of disease were present even during the **first stage** whereas signs of illness were typically present in the **second** and **third stages**. Two common features emerged: the level of pain was worse than anything previously experienced, and painkillers had no effect. The patients were unable to articulate their sensation of pain: *“The pain increased … it became unbearable and I was just screaming from pain when I came to the hospital…” … “The pain was indescribable, so violent and completely extreme”.* Uniquely for the patients (6 men and 1 woman) with an infection in the genitals/groin, the infection presented itself as a boil or a small abscess. The pain intensity in this area was not described as unbearable.

**Table 3:** Patient experience of symptoms and family experience of signs r/t NSTI

Symptoms (reported by patient)	Signs (reported by patient and family)
Pain (*n* = 20) Feeling of being unwell Chills or shivering Flu-like symptoms, aching body Nausea Decreased mobility Tired, fatigued, weak Abnormal thirst Feeling that something is very wrong Anxiety, fear, panic Difficulty breathing Dizzy or confused Apathy or listlessness	High fever (*n* = 14) Local swelling (*n* = 13) Red or blue skin (*n* = 12) Vomiting or diarrhea (*n* = 9) Boil or abscess (*n* = 7) Chills or fever (*n* = 5) Rapid heart rate (*n* = 3) Local heat (*n* = 2)

### Types of diagnostic delay

Most of the participants experienced diagnostic delay. We have categorized delay as related to the patient, primary care and hospital care.

#### Diagnostic delay related to the patient

Patient/family related delays were uncommon, but delay was seen in a few of our participants for the following reasons: too weak to visit the ED, expecting it to get better, forgetting an appointment with the GP or the regular GP unavailable. Initial contact was typically to the on-call or medical service. Otherwise the patients usually made an appointment with the primary care center or their GP.

#### Diagnostic delay related to primary care

According to the narratives, the main reasons for nurse/physician delays were:

1. Misdiagnosis such as the flu, a sore throat or a stomach virus.
2. Misdiagnosis related to lack of acknowledgment of the patient’s or family’s complaints: *“I felt uneasy, weak, dizzy, had no appetite and had high fever and also heart palpitations and couldn’t move… we called the primary care center and medical on-call service several times over the course of 3 days…”* Wife*: “He was not himself, he was in such pain and throwing up but they all said that it was completely normal to feel this way…”*

Patients that contacted the primary care center were advised to go to the hospital, but an ambulance was not dispatched. Among our participants, 18 arrived at the hospital by car or public transportation, 6 by ambulance, and two unspecified. Three patients requested an ambulance, but this was denied. One patient waited for 4 h for transportation and finally reached the hospital in a critical state:

[Family] “*When he arrived at the hospital he had no blood pressure … they thought that there must be something wrong with the machine….”*

The families were expected to drive the patient to the nearest hospital, even if the patient was unable to walk:

*“She supported me all the way to the car, put me in the front seat, fastened my seatbelt, when I just tumbled over to her side”.*

*“I was in extreme pain, my heart rate was over 170, it was difficult to move and to get into the car….”*

#### Diagnostic delay related to the hospital

Delays at the hospital were usually due to waiting in the ED. Patients waited for 2–8 h if the triage nurse failed to recognize the gravity of the situation:

*“There was no one in the ED checking on him for 3 hours… I could see he was just getting worse and worse…”.*

Other reasons for hospital related diagnostic delay were e.g. premature diagnosis (erysipelas, hepatitis, kidney failure, gallbladder rupture, heart failure) and missed or overlooked important clues towards correct diagnosis. Several family members described their despair when unable to draw attention to what they thought was important or wrong. Many participants described how they were not taken seriously, and how this led to unnecessary delays:

*“He could not sit so I tried to support him ... I had to ask for a bed and then he had to lie there in a hallway. He was so thirsty and shivering and in such pain; I tucked him in with my jacket and had to ask the nurses to give him painkillers… they said that a doctor would check on him in some hours … He was so afraid, I asked the nurse assistant if he could get a sedative to ease this terrible experience … but she answered that this was not so bad….”*

*“I said to a nurse* [*at the ED*] *‘please, please can someone look at my husband’s foot’…but no, there was no doctor there that had the time”.*

## Discussion

The aim of our study was to explore the patient and family experience of early signs and symptoms of NSTI and to describe the initial contact with the healthcare system. Our main findings were the detailed descriptions of long, moderate and short inception, providing an illustration of the variation in duration of antecedent signs and symptoms. Other important findings included the description of three stages of early disease progression with increase in symptom intensity. Finally, our study suggested that the level of pain experienced in NSTI is particularly excruciating and unresponsive to over-the-counter pain medications.

Our study refutes the typical description of disease progression in the literature as always rapid [1, 3]. According to the patients, the antecedent signs and symptoms prolonged the disease experience, albeit the disease progression was in fact rapid after diagnosis of NSTI. The varying inception periods and the subjective symptoms were described in detail by the participants in our study. They had a strong feeling that something was very wrong when patients developed symptoms such as dyspnea, shivering, muscle weakness, gastrointestinal problems, and altered mental status.

The patients in our study all experienced a number of symptoms as a precursor to NSTI. Pain is the main symptom described in the current literature [1, 2, 16], albeit it is not considered a diagnostic variable because it is non-specific. Current recommendations rely on quantifiable signs and late clinical findings such as the “positive fingers test” (probing fascia to see if tissue dissects), dishwasher pus and/or grey necrotic tissue [16, 27, 28]. Wong et al. [29] have developed a risk index score, Laboratory Risk Indicator for Necrotizing infections (LRINEC) to distinguish NSTI from non-necrotizing infections. The NSTI guidelines, however, state that the LRINEC score lacks the sensitivity to be useful [2]. In contrast, a recent systematic review demonstrated a positive correlation between LRINEC score and a true diagnosis and concluded that the scoring system is a useful clinical determinant in the diagnosis of NSTI [30]. Our study suggests that the LRINEC score might not be relevant in the early detection of disease since abnormal values are also late signs of severe infections. Patient reported symptoms are often described before quantifiable signs of infection, presenting an argument for the inclusion of the patient’s narrative as part of the diagnostic process. We suggest that patient and family narratives and description of antecedent signs and symptoms should be included more systematically in the primary care and emergency settings. This would make sense as emerging studies suggest that patient participation might prevent adverse events [31]^.^

Other research has focused on the diagnostic value of symptoms. A study by Edman-Wallér et al. [32] shows that altered mental status and dyspnea are symptoms associated with severe sepsis. As diagnosis of NSTI is and probably will remain difficult, a structured use of common symptoms in combination with signs seems to be a promising way towards reducing delays. It is important to stress that experiencing NSTI is traumatizing, and not listening to patients or family members, neglecting them or not involving them in the care might increase patient suffering and diagnosis delay. On the other hand, being taken seriously and met with empathy and respect seems to ease the emotional pain.

Our study illustrates how delays occur during the early stages of NSTI for various reasons. Some delays were patient related and are, perhaps, difficult to change. Other delays were seen within the healthcare system where there might be room for improvement. We believe that the patient and family perspective was in some cases ignored when the patient presented symptoms of infection. It was surprising that patients with very high CRP and signs of severe illness at primary care centers were told to make their own way to the ED instead of dispatching an ambulance. We assume the severity of illness was not recognized.

Our findings demonstrate that patients with NSTI risked misdiagnosis when healthcare professionals prematurely decided on an incorrect diagnosis. NSTI requires quick diagnosis and treatment, but premature diagnosis might prevent correct diagnosis when more data are available [30]. It is also common to go for the most obvious and overlook other explanations, as we have demonstrated in several of our cases. However, specific training strategies exist that can be applied to minimize diagnostic error, including awareness of cognitive and contextual factors that predispose for diagnostic errors, and simulation training of diagnostic reasoning [33]. Aside from that, there seems to be a need for better basic knowledge about the diverse manifestations of NSTI among nurses and physicians in primary healthcare but also among health professionals working in the ED. We hope our findings will increase awareness of antecedent signs and symptoms of NSTI and subsequently prevent misdiagnosis and diagnostic delay. We suggest the integration of patient reported symptoms and guidelines with objective measures. There is a need for studies describing whether symptoms and symptom clusters can assist diagnosis and prevent fatal outcomes. We believe that a campaign like the *Surviving Sepsis Campaign* (http://www.survivingsepsis.org) is a good example of an initiative that provides guidelines, educational material and recommendation for implementation that could inspire help to spread awareness and knowledge about NSTI in the society.

### Strengths and limitations

The credibility of our study was increased by the adoption of well-established research methods and investigator triangulation. We encouraged the participants to be frank in their responses and used iterative questioning to increase transparency. As such, our study generated important new knowledge based on subjective experiences of NSTI that cannot be measured quantitatively. A qualitative design does not allow for generalizations, thus the findings cannot be applied directly to a wider population or to other settings and populations. However, we assume that there is transferability to other similar settings. Our study generated hypotheses that could be tested in a larger-scale investigation with a quantitative design to enable generalization. We realize there is potential survivor-bias in our study and we acknowledge that non-survivors’ experiences might differ from our findings. Furthermore, as our investigation was limited to streptococcal, microbial and polymicrobial infections, future studies exploring the early development in relation to anaerobe microorganisms could contribute with important information. Recall-bias is a limitation in qualitative interview studies [34]. We had to balance the scientific advantage of early interviewing with the ethical disadvantages of premature infringement on the participants. We discussed this matter with the NSTI-survivor involved in the planning of the study and decided on interviewing about 6 months post diagnosis. We increased the trustworthiness of our study by interviewing both patient and family.

## Conclusion

Our study adds to the understanding of the lived experience of NSTI by providing in-depth description of antecedent signs and symptoms precipitating NSTI-diagnosis. Inception periods vary from over a week to less than a day, while symptoms escalate in severity increasing frustration and fear in patient and family. We described diagnostic delay as patient-related, primary care related, or hospital related and recommend that patient and family narratives should be considered when diagnosing NSTI to decrease diagnostic delay.

## Abbreviations

CRP: C-reactive protein
ED: Emergency department
ER: Emergency room
NSTI: Necrotizing soft tissue infection
